# AGING AND LONGITUDINAL EFFECTS ON NUMERACY

**DOI:** 10.1093/geroni/igac059.2253

**Published:** 2022-12-20

**Authors:** Ryan Best, Katherine Carman, Andrew Parker

**Affiliations:** West Virginia University, Morgantown, West Virginia, United States; RAND Corportation, Santa Monica, California, United States; RAND Corporation, Pittsburgh, Pennsylvania, United States

## Abstract

Numeracy, the ability to competently make use of numbers and numerical information, is a skill associated with diverse positive outcomes across the lifespan. Numeracy is related to increases in education attainment, economic success, and the quality of health and financial decisions (e.g., Chesney, Bjälkebring, & Peters, 2015; Reyna et al., 2009; Smith, McArdle, & Willis, 2010). More generally, numeracy correlates positively with measures of fluid and crystallized intelligence, but accounts for a unique portion of the variance in models predicting risk comprehension (Cokely et al., 2012) and performance in many decision-making tasks (e.g., Peters et al., 2006; Peters, 2012). Age effects on cognitive functioning are well established, generally describing declines in fluid abilities and increases or stability in crystallized abilities (for a review, see Salthouse 2010), but little is known about the longitudinal trajectory of numeracy into older age. The current study investigates longitudinal age effects on numeracy using a sample of 524 adults (2008 Agerange = 20-78) from the RAND American Life Panel. Participants completed a numeracy measure in both 2008 and 2019, a span of 11 years. Results show that numeracy scores generally decreased between measurement points (β = -.24, t = -2.27, p = .03) and that increased age was predictive of a larger decrease in numeracy scores (β = -.02, t = -2.07, p = .04). Results are discussed as they relate to cognitive aging and how the trajectory of numeracy compares to other cognitive constructs.

